# Paraoxonase1 Genetic Polymorphisms in a Mixed Ancestry African Population

**DOI:** 10.1155/2014/217019

**Published:** 2014-11-16

**Authors:** M. Macharia, A. P. Kengne, D. M. Blackhurst, R. T. Erasmus, T. E. Matsha

**Affiliations:** ^1^Division of Chemical Pathology, Faculty of Health Sciences, National Health Laboratory Service (NHLS) and University of Stellenbosch, Cape Town 7505, South Africa; ^2^Non-Communicable Diseases Research Unit, South African Medical Research Council, University of Cape Town, Cape Town 7505, South Africa; ^3^Division of Chemical Pathology, University of Cape Town, Cape Town 8000, South Africa; ^4^Department of Biomedical Sciences, Faculty of Health and Wellness Science, Cape Peninsula University of Technology, P.O. Box 1906, Bellville, Cape Town 7530, South Africa

## Abstract

Paraoxonase 1 (PON1) activity is markedly influenced by coding polymorphisms, Q/R at position 192 and M/L at position 55 of the PON1 gene. We investigated the frequencies of these polymorphisms and their effects on PON1 and antioxidant activities in 844 South African mixed ancestry individuals. Genotyping was done using allele-specific TaqMan technology, PON1 activities were measured using paraoxon and phenylacetate, oxidative status was determined by measuring the antioxidant activities of ferric reducing antioxidant power and trolox equivalent antioxidant capacity, and lipid peroxidation markers included malondialdehyde and oxidized LDL. The frequencies of Q192R and L55M were 47.6% and 28.8%, respectively, and the most common corresponding alleles were 192R (60.4%) and 55M (82.6%). The *Q192* was significantly associated with 5.8 units' increase in PON1 concentration and 15.4 units' decrease in PONase activity after adjustment for age, sex, BMI, and diabetes, with suggestion of differential effects by diabetes status. The PON1 *L55* variant was associated with none of the measured indices. In conclusion, we have shown that the Q192R polymorphism is a determinant of both PON1 concentration and activity and this association appeared to be enhanced in subjects with diabetes.

## 1. Introduction

Paraoxonase 1 (PON1) is a calcium dependent esterase synthesized in the liver and widely distributed in tissues including liver, kidney, intestine, and serum, where it associates with high-density lipoprotein (HDL). The enzyme has a dual physiological function in humans. First, it catalyzes the breakdown of various toxic organophosphate (OP) pesticides and nerve gases, including paraoxon, diazoxon, sarin, and soman [1, 2], which are potent acetylcholinesterase (AChE) inhibitors. Secondly, PON1 is increasingly acknowledged as an atheroprotective enzyme due to its in vitro ability to inhibit oxidative modifications of LDL [3], HDL [4], macrophages [5], atherosclerotic lesions [6], and augment cholesterol efflux from macrophages [7]. In addition, PON1 lowers inflammatory responses in the arterial wall by destroying biologically active lipids in mildly oxidized LDL [8], impairing the differentiation of monocytes to macrophages [9], and decreasing monocyte chemotaxis and adhesion to endothelial cells [10]. Decreased PON1 activities have been reported in diseases with accelerated atherogenesis including diabetes and familial hypercholesterolemia [11–13].

The activity of the enzyme is markedly influenced by polymorphisms on the coding and promoter regions of the PON1 gene. The coding polymorphisms are Q/R at position 192 and M/L at position 55 which result in isozymes differing greatly in their activity toward various substrates [2, 14, 15]. The R isoform hydrolyzes paraoxon faster than the Q isoform, whereas diazoxon, soman, and sarin are hydrolyzed at a higher rate by the Q than R isoform [2]. In contrast, the R isoform is less effective at hydrolyzing lipid peroxides than the Q isoform [2]. The M and L alleles are associated with lower and higher serum PON1 concentrations, respectively [16]. The distribution of the Q192R and L55M polymorphisms widely varies worldwide. For example, the frequency of the* PON1* Q192 allele has a high frequency in Caucasians (0.70) [17, 18], but a considerably lower frequency in Mexicans (0.48) [19] and African-Americans (0.34) [18]. The* PON1* L55 allele predominates in nearly all populations but variations still exist, for example, between Taiwanese (0.97) [20], Gabonese (0.695) [21], Turkish (0.39) [22], and Iranians (0.59) [23].

This study was undertaken to investigate the frequencies of* PON1* Q192R and L55M polymorphisms and their possible relationship with PON1 activity and indices oxidative status (ferric reducing antioxidant power, trolox equivalent antioxidant capacity, malondialdehyde, and oxidized LDL). Herein, we investigated the mixed ancestry population from South Africa that has been shown to have one of the highest prevalence of type 2 diabetes in South Africa and sub-Saharan Africa at large [24].

## 2. Materials and Methods

### 2.1. Study Setting and Population

Details of the study including survey design and procedures have been described elsewhere [24, 25]. Study participants were members of a cohort study conducted in a mixed ancestry township (Bellville South) which is located within the Northern suburbs of Cape Town, Western Cape, South Africa. The mixed ancestry population of South Africa, sometimes referred to as “coloured,” is of mixed genetic origin with contributions from Europeans, South Asians, Indonesians, and a population genetically close to the isiXhosa sub-Saharan Bantu [26]. The study was approved by the research ethics committees of Stellenbosch University (reference number: N10/04/118) and the Cape Peninsula University of Technology (CPUT/HW-REC 2010/H017) and was conducted according to the Code of Ethics of the World Medical Association (Declaration of Helsinki). All participants signed written informed consent after all the procedures were fully explained in the language of their choice. All participants received a standardized interview and physical examination during which blood pressure was measured according to the World Health Organisation (WHO) guidelines [27] using a semiautomated digital blood pressure monitor (Rossmax PA, USA) on the right arm in a sitting position. Anthropometric measurements were performed three times and their average used for analysis: weight (kg), height (cm), and waist (cm) and hip (cm) circumferences. Participants with no history of doctor diagnosed diabetes mellitus underwent a 75 g oral glucose tolerance test (OGTT) as recommended by the WHO [28]. Further, the following biochemical parameters were determined on the Cobas 6000 Clinical Chemistry instrument (Roche Diagnostics, Germany): fasting plasma glucose, insulin, total cholesterol (TC), high density lipoprotein cholesterol (HDL-c), triglycerides (TG), C-reactive protein (CRP), *γ*-glutamyltransferase (GGT), and glycated haemoglobin (HbA1c) certified by National Glycohaemoglobin Standardisation Programme (NGSP). Low density lipoprotein cholesterol (LDL-c) was calculated using Friedewald's formula [29]. Serum cotinine was measured by chemiluminescent assay (Immulite 1000, Siemens).

### 2.2. Total Antioxidant Capacity

The total antioxidant capacity in plasma samples was assessed using the ferric reducing antioxidant power (FRAP) and trolox equivalent antioxidant capacity (TEAC) assays. FRAP was done according to the method of Benzie and Strain [30]. Briefly, plasma samples were mixed with FRAP reagent, incubated for 30 min at 37°C, and the absorbance at 593 nm was recorded using a spectrophotometer (Spectramax plus384 Molecular devices, USA). The TEAC assay was according to Re et al. [31] and is based on monitoring (at 734 nm) the oxidation of 2,2′-azinobis-(3-ethylbenzothiazoline-6-sulfonic acid) radical (ABTS) cation formed by reacting ABTS and potassium persulfate. Distilled water was used instead of PBS to dilute the ABTS^+^ radical solution.

### 2.3. Paraoxonase Activity

Paraoxonase (PONase) and arylesterase (AREase) activities were measured using paraoxon and phenylacetate (Sigma Aldrich, SA) as substrates, respectively. PONase activity was measured using the method of Richter and Furlong [32] from the initial velocity of p-nitrophenol production at 37°C and the increased absorbance at 405 nm was monitored on a spectrophotometer (Spectramax plus384, Molecular devices, USA). Each serum sample was incubated with 5 mmol/L eserine (Sigma Aldrich, SA) for 15 minutes at room temperature to inhibit serum cholinesterase activity which is usually elevated in diabetes and would otherwise interfere with the determination of paraoxonase activity in serum from diabetic individuals. PON-1 activity of 1 U/L was defined as 1 *μ*mol of p-nitrophenol hydrolyzed per minute. A slightly modified method of Browne et al. [33] was used to measure AREase activity. The working reagent consisted of 20 mmol/L Tris-HCl, 4 mmol/L phenyl acetate, pH 8.0, with 1.0 mmol/L CaCl_2_ (Sigma Aldrich, SA). The reaction was initiated by adding 5 *μ*L of 40-fold tris-diluted samples to 345 *μ*L of the working reagent at 25°C. The change in absorbance at 270 nm was recorded for 60 minutes after a 20-second lag time on a Spectramax plus384 spectrophotometer. The activity, expressed as kU/L, was based on the molar absorptivity (1310) of phenol at 270 nm. In both assays, the rates used to generate the data points were derived from the linear portions of the rate versus time plots.

### 2.4. Lipid Peroxidation

Plasma MDA and ox-LDL were used as markers of lipid peroxidation (LPO). The method of Jentzsch et al. [34] was used to estimate the thiobarbituric acid reactive substances (TBARS) which reflect the production of MDA. Plasma ox-LDLs were measured using a quantitative sandwich ELISA kit (Cellbiolabs, San Diego, California).

### 2.5. Genotyping

DNA was extracted from whole blood using the salting-out method of Miller et al. [35]. Conventional polymerase chain reaction (PCR) followed by direct DNA sequencing was performed for detection of the wild type, heterozygous, and homozygous genotypes of PON1 single nucleotide polymorphisms (SNPs),* Q192R* (rs662, A>G), and* L55M* (rs854560, T>A). These internal control samples were subsequently used for analytical validation of high throughput genotyping performed on DNA samples extracted from the study participants, using the Applied Biosystems (ABI)* Taq*Man SNP Genotyping Assays on the ABI Prism 7900HT platform (Applied Biosystems, USA).

### 2.6. Definitions

Body mass index (BMI) was calculated as weight per square meter (kg/m^2^) and waist-hip-ratio (WHR) as waist/hip circumferences (cm). Type 2 diabetes status was based on a history of doctor-diagnosis, a fasting plasma glucose ≥7.0 mmol/L, and/or a 2-hour post-OGTT plasma glucose ≥11.1 mmol/L. The homeostatic model assessment of insulin resistance (HOMA-IR) was calculated according to the following formula: HOMA-IR = [fasting insulin concentration (mIU/L) × fasting plasma glucose (mmol/L)]/22.5; while functional *β*-cells (HOMA-B%) were estimated using the formula: 20 × fasting insulin (*μ*IU/mL)/fasting glucose (mmol/mL) − 3.5. The quantitative insulin-sensitivity check index (QUICKI) as 1/[log(fasting insulin (*μ*U/mL)) × log(fasting glucose (mg/dL))].

### 2.7. Statistical Methods

Of the 946 participants who took part in the survey, 941 consented for genetic studies. Among the latter, 103 were excluded for missing data on the genetic variables. Oxidative status profile was assessed in 491 subjects, but 121 were also excluded on account of missing consent or fully matching biochemical and genetic data. Therefore, 844 and 370 subjects had valid data for the overall genetic and genetic-oxidative stress analyses, respectively. General characteristics of the study participants are summarized as count and percentage for dichotomous traits, mean and standard deviation (SD), or median and 25th–75th percentiles for quantitative traits. Traits were log-transformed to approximate normality, where necessary, prior to analysis. SNPs were tested for departure from Hardy-Weinberg Equilibrium (HWE) expectation via a chi square goodness of fit test. Linkage disequilibrium (LD) was estimated using the D′ statistic. The chi square analysis of the variance (ANOVA) and Kruskal Wallis test were used to compare baseline characteristics across allele's distribution. The interaction between SNPs was assessed through robust linear regression models, assuming additive models for the SNPs. Results corresponding to *P* values below 5% are described as significant. We did not adjust for multiple testing. All analyses used the statistical package R (version 3.0.0 [2013-04-03], The R Foundation for statistical computing, Vienna, Austria).

## 3. Results

### 3.1. Distribution of PON1 Polymorphisms

Of the 844 subjects (men 208, 24.6%) 238 (28.2%) had diabetes, 478 (56.6%) were hypertensive, and the average age was 56.6 (15.5) years. Genotype and allele frequencies for the L55M and Q192R polymorphisms in the overall cohort as well as across genders and diabetes status are summarized in Table 1. The frequencies of Q192R and L55M were 47.6% and 28.8%, respectively, and the most common corresponding alleles were 192R (60.4%) and 55 M (82.6%). Frequencies for both polymorphisms were not different between major subgroups, except for the Q192R genotype which differed significantly according to diabetes status (*P* = 0.028). Observed and expected frequencies for both polymorphisms were in Hardy-Weinberg equilibrium overall and within major subgroups (all *P* ≥ 0.085). The linkage between the two polymorphisms was moderate in the overall sample (D′ = 0.470, *P* < 0.0001) but comparatively weaker in men (D′ = 0.199, *P* = 0.0001), women (D′ = 0.290, *P* < 0.0001), and in participants with (D′ = 0.219, *P* < 0.0001) or without diabetes (D′ = 0.283, *P* < 0.0001).

### 3.2. Baseline Profile Overall and Across PON1 Genotypes

The distribution of the baseline characteristics was not different within the various PON1 genotypes with respect to age, sex, adiposity, lipid profile prevalence of hypertension, and insulin resistance (Table 2). This, however, was not the case for prevalent diabetes (*P* = 0.028) and systolic blood pressure levels (*P* = 0.04) across Q192R polymorphism, and fasting plasma glucose (*P* = 0.019), 2 hr glucose (*P* = 0.038), and HbA1c (*P* = 0.004) across L55M polymorphism (Table 2).

### 3.3. PON1 and Oxidative Status Profile Across Genotypes

Table 3 shows the distribution of indices of PON1 and antioxidant activities across the genotypes. PON1 (*P* = 0.015), PONase (*P* < 0.0001), Ox-LDL (*P* = 0.029), TBARS (*P* = 0.006), and to some extent TEAC (*P* = 0.053) were significantly different across genotype of PON1 Q192R polymorphism, whereas fasting glucose (*P* = 0.019), 2-hour glucose (*P* = 0.038), and HbA1c (*P* = 0.0004) varied across PON1 L55M genotypes.

Table 4 shows the results from robust linear regression analyses for the prediction of PON1 and antioxidant status indices by the two PON1 variants. The PON1 Q192 polymorphism was significantly associated with PON1 concentration and PONase activity resulting in unit increases of 6.8 and decreases of 18.3, respectively. The association remained significant when the models were expanded stepwise to include age, sex, BMI, and diabetes with only modest attenuation of the effect size. However, when the interaction term of diabetes and Q192 was added to multivariable models, the main effect of the polymorphism remained significant for the prediction of PONase but was substantially attenuated for PON1 concentration with a borderline association (*β* = 3.79, *P* = 0.08). Furthermore, the effect of the interaction term diabetes^*^Q192 was significant for the prediction of PON1 concentration (interaction *P* = 0.007), but not for PONase, suggesting that the effect of the variant on PON1 concentration was more important in participants with diabetes (Table 4). Alone or with the other covariates in the models, the PON1 L55 was not significantly associated with any of the measured indices. However, there was a suggestion of a significant interaction by diabetes status in the effect of PON1 L55 on ox-LDL levels (interaction *P* = 0.013), with suggestion of a positive effect on ox-LDL levels in participants with diabetes and a negative or no effect in participants without diabetes (*β* = −395.49, *P* = 0.075) for the main effect of the variant in the multivariable model containing the interaction term gene^*^diabetes. In the polymorphisms only model (with and without their interaction term), the effects of variants on PON1 concentration and PONase remained significant for PON1 Q192 and nonsignificant for PON1 L55. Effects on other analytes remained unchanged with always no evidence of Q192^*^L55 polymorphic interaction (Table 4).

## 4. Discussion

Paraoxonase 1 polymorphisms Q192R and L55M have been reported to explain over 90% of total phenotypic variance in PON1 activity using several substrates of the enzyme [36]. In this study, we used paraoxon and phenylacetate substrates to characterize the influence of both Q192R and L55M PON1 polymorphisms on PON1 concentrations and enzyme activity in a mixed ancestry population from South Africa. Only the Q192R appeared to be functional in this population as it was associated with both PON1 concentration and the paraoxonase activity. We observed that the presence of Q192 was associated with 15.4 U/L decrease and 5.8 *μ*g/mL increase in PON1 activity and concentration after accounting for the effects of age, sex, BMI, and diabetes. There was indication that the effect of the variant on PON1 concentration was more important in participants with diabetes. In parallel, we report PON1 QQ192 to be associated markers of oxidative stress (ox-LDL and TBARS) and total antioxidants (TEAC) only in cross-genotype comparison, but not in linear regression (irrespective of the level of adjustment), possibly suggesting the absence of a relationship, the nonlinearity of the association if any, or the inadequacy of the log-additive model to approximate such an association.

PON1 activity can be measured using different substrates and reports have shown that the effect of PON1 polymorphisms varies according to the substrate used [2, 14, 15]. Among three of the commonly used substrates (paraoxon, phenyl acetate, and dihydrocoumarin), the most pronounced genotype effects were for PON1 paraoxon [36]. Previous studies have associated the R allele with higher risk and/or incidence of atherosclerotic heart disease in various populations such as Indians [37], Japanese [38], and Dutch [39]. On the other hand, Bhattacharyya et al. [40] conducted a prospective study of 1399 patients and reported higher serum levels of PON1 activity, lower systemic indices of systemic oxidative stress, and corresponding reductions in both prevalent coronary artery disease and prospective cardiac events in PON1 192RR carriers. Similarly, the findings of the present study appear to suggest a decreased atherosclerotic risk in subjects with PON1 192R since the presence in the PON1 Q192 was significantly associated with reduction of PON1 activity which in turn has been associated with the development of CVD [41, 42]. Furthermore, PON1 QQ192 genotype was associated with increased PON1 concentration especially in subjects with diabetes. Our results could perhaps be explained by the different effect of the genotypes on HDL-bound PON1 since PON1 has to be bound to HDL to perform its antiatherosclerosis function [4]. The Q192 alloenzyme binds to the HDL particle with 3-fold lower affinity than the R192 alloenzyme [43], but the HDL-bound QQ192 PON1 has been shown to be more effective at protecting the oxidation of LDL [2, 44]. Furthermore, it has been shown that increased HDL-bound PON1 content does not alter the HDL composition or properties but protects it from lipid peroxidation [4]. Taken together, our results show that the R allele increases PON1 activity but also indicate that the PON1 QQ192 may be important in individuals with increased oxidative stress such as diabetes even though it suppresses the activity of the enzyme. Our findings, however, need to be confirmed in prospective studies with a larger sample size.

In our study we also show the predominance of PON1 55M (82.6%) in this population, an unusual finding reported in only one other study in much lower proportion (61%) [22]. The PON1 L55 PON1 has been associated with increased PON1 activity [45]; however, this was not apparent in this study. It is however worth noting that the distribution of indices of glycaemic control (FBG, 2 hr glucose, and HbA1c) differs significantly across the L55M genotypes which may suggest an association with poorer glucose control and therefore glycation-enhanced oxidative stress. Although we had a relatively large population for robust linear regression studies based on the number of predictors assessed in the current study, it is likely that the low frequency of some genotypes (in PON1 L55M in particular) has affected our power for uncovering some significant associations. However, our study also has major strengths. Unlike many existing studies, in addition to PON1 polymorphisms, we measured PON1 protein levels and activity by two methods and assessed oxidative status via several methods to demonstrate the consistency of our results.

In conclusion, we have shown that the Q192R polymorphism is a determinant of both PON1 concentration and activity. This association appeared to be enhanced in subjects with diabetes, thus suggesting a need to adjust for potential genetic confounding in future PON1 studies, involving diabetic subjects.

## Figures and Tables

**Table 1:** Genotype distributions, minor allele frequencies, and unadjusted *P* values for comparing genotype distributions according to diabetes status, and additive allelic effects between diabetes groups.

	Without diabetes	Diabetes	*P*	Overall	Men	Women	*P*
*N*	606	238		844	208	636	

PON1 rs662							
QQ192, *n* (%)	97 (16.0)	36 (15.1)	0.028	133 (15.8)	32 (15.4)	101 (15.9)	0.722
Q192R, *n* (%)	272 (44.9)	130 (54.6)		402 (47.6)	104 (50.0)	298 (46.9)	
192RR, *n* (%)	237 (39.1)	72 (30.3)		309 (36.6)	72 (34.6)	237 (37.3)	
Q, *n* (%)	466 (38.4)	202 (42.4)		668 (39.6)	168 (40.4)	500 (39.3)	
R, *n* (%)	746 (61.6)	274 (57.6)		1020 (60.4)	248 (59.6)	772 (60.7)	
HWE (*P* value)	0.199	0.085		0.943	0.666	0.678	

PON1 rs854560							
55MM, *n* (%)	420 (69.3)	156 (65.6)	0.168	576 (68.3)	152 (73.1)	424 (66.7)	0.066
L55M, *n* (%)	172 (28.4)	71 (29.8)		243 (28.8)	54 (26.0)	189 (29.7)	
LL55, *n* (%)	14 (2.3)	11 (4.6)		25 (3.0)	2 (1.0)	23 (3.62)	
L, *n* (%)	200 (16.5)	93 (19.5)		293 (17.4)	58 (13.9)	235 (18.5)	
M, *n* (%)	1012 (83.5)	383 (80.5)		1395 (82.6)	358 (86.1)	1037 (81.5)	
HWE (*P* value)	0.556	0.412		>0.999	0.383	0.694	

HWE: Hardy-Weinberg Equilibrium (HWE *P* values are from exact tests).

**Table 2:** Genes and baseline characteristics.

Characteristics	PON1 rs662				PON1 rs854560				Overall	Int *P*
	QQ192	Q192R	192RR	*P* value	55MM	L55M	LL55	*P* value		
*n*	133	402	309		576	243	25		844	
Men, *n* (%)	32 (24.1)	104 (25.9)	72 (23.3)	0.722	152 (26.4)	54 (22.2)	2 (8)	0.066	208 (24.6)	0.239
Diabetes, *n* (%)	36 (27.1)	130 (32.3)	72 (23.3)	0.028	156 (27.1)	71 (29.2)	11 (44)	0.168	238 (28.2)	0.243
Hypertension (%)	76 (57.1)	240 (59.7)	162 (52.4)	0.151	329 (57.1)	135 (55.6)	14 (56)	0.917	478 (56.6)	0.285
Age, years (SD)	55.4 (15.0)	55.2 (15.0)	52.6 (16.2)	0.052	55.0 (15.6)	54.1 (15.5)	61.4 (12.0)	0.063	54.3 (15.5)	0.078
Systolic blood pressure (mmHg)	127 (23)	126 (21)	122 (20)	0.040	124 (20)	126 (22)	125 (23)	0.0672	125 (20)	0.703
Diastolic blood pressure (mmHg)	76 (17)	76 (12)	74 (12)	0.138	75 (13)	76 (13)	74 (15)	0.433	76 (13)	0.126
Body mass index (kg/m^2^)	29.4 (7.1)	29.9 (7.1)	29.4 (7.0)	0.569	29.4 (7.0)	30.2 (7.3)	30.3 (7.3)	0.288	29.6 (7.1)	0.931
Waist circumference (cm)	97 (15)	96 (15)	96 (16)	0.649	96 (15)	97 (17)	98 (13)	0.482	96 (15)	0.091
Hip circumference (cm)	109 (15)	109 (14)	108 (14)	0.763	108 (14)	110 (14)	110 (12)	0.527	109 (14)	0.695
Waist/hip ratio	0.89 (0.09)	0.88 (0.08)	0.88 (0.11)	0.875	0.88 (0.08)	0.89 (0.12)	0.89 (0.08)	0.785	0.88 (0.10)	0.056
Fasting plasma glucose (mmol/L)	6.5 (2.9)	6.6 (3.3)	6.3 (3.4)	0.385	6.3 (2.9)	6.7 (3.8)	8.0 (4.7)	0.019	6.5 (3.3)	0.105
2-hour glucose (mmol/L)	7.2 (2.6)	7.7 (3.9)	7.3 (3.4)	0.343	7.4 (3.3)	7.5 (3.4)	9.6 (7.9)	0.038	7.5 (3.5)	0.762
HbA1c (%)	6.3 (1.5)	6.4 (1.5)	6.3 (1.6)	0.641	6.2 (1.4)	6.4 (1.6)	7.4 (2.6)	0.0004	6.3 (1.5)	0.170
Total cholesterol (mmol/L)	5.6 (1.2)	5.6 (1.2)	5.5 (1.2)	0.358	5.6 (1.2)	5.6 (1.2)	5.9 (1.2)	0.372	5.6 (1.2)	0.373
HDL cholesterol (mmol/L)	1.3 (0.4)	1.3 (0.3)	1.3 (0.4)	0.693	1.3 (0.4)	1.3 (0.3)	1.4 (0.4)	0.082	1.3 (0.4)	0.857
Triglycerides (mmol/L)	1.5 (1.0)	1.5 (0.9)	1.5 (1.0)	0.843	1.5 (0.9)	1.5 (1.0)	1.5 (0.7)	0.885	1.5 (0.9)	0.141
LDL cholesterol (mmol/L)	3.7 (1.0)	3.7 (1.0)	3.6 (1.0)	0.457	3.6 (1.0)	3.7 (1.0)	3.8 (1.1)	0.792	3.6 (1.0)	0.723
Gamma glutamyl transferase (IU/L)	27 [19–40.2]	26 [18–39]	26 [18–42.2]	0.748	27 [18–41]	25 [18–36]	30 [21–49]	0.321	27 [18–40]	0.325
C-reactive protein (mg/L)	3.5 [1.3–7.6]	4.2 [1.1–9.8]	3.5 [1.1–8.1]	0.446	3.9 [1.1–8.8]	3.5 [1.1–8.7]	5.8 [2.7–11.0]	0.165	3.8 [1.1–8.8]	0.456
Cotinine (ng/mL)	10 [10–251]	10 [9–247]	10 [10–341]	0.073	10 [9–309]	10 [9.7–247.5]	10 [9-10]	0.080	10 [9–283]	0.928
Fasting insulin (*μ*U/mL)	8 [3.7–14.1]	7.3 [3.3–13]	7.1 [2.7–13.9]	0.667	7.4 [2.9–13.7]	7.6 [3.7–13.8]	6.8 [3–7.5]	0.389	7.4 [3.1–13.5]	0.752
2 h insulin (*μ*U/mL)	35.7 [16–84.6]	36.7 [20–60.7]	34.7 [15.6–73.1]	0.890	35.7 [18.7–66.7]	36.6 [17–69.9]	29.6 [13.8–56.7]	0.660	36.3 [17.5–68]	0.033
Median glucose/insulin ratio (*μ*U/mL)	0.76 [0.39–1.58]	0.79 [0.48–1.79]	0.74 [0.42–1.81]	0.718	0.79 [0.42–1.81]	0.72 [0.45–1.51]	0.89 [0.71–2.74]	0.151	0.77 [0.43–1.77]	0.462
Median HOMA-IR	2.1 [1.0–3.8]	1.8 [0.9–1.7]	1.8 [0.6–3.4]	0.302	1.8 [0.7–3.8]	1.9 [0.9–3.6]	1.9 [0.7–2.7]	0.756	1.8 [0.7–3.7]	0.540
Median HOMA-B%	76 [24–158]	63 [25–124]	77 [27–149]	0.150	70 [23–141]	71 [33–142]	56 [15–104]	0.248	69 [25–138]	
Median QUICKI	0.15 [0.14–0.17]	0.15 [0.14–0.17]	0.15 [0.14–0.18]	0.354	0.15 [0.14–0.18]	0.15 [0.14–0.17]	0.15 [0.14–0.17]	0.694	0.15 [0.14–0.17]	0.079
Median 1/HOMA-IR	0.48 [0.26–1.05]	0.56 [0.27–1.16]	0.57 [0.29–1.63]	0.302	0.55 [0.26–1.46]	0.53 [0.27–1.10]	0.51 [0.37–1.32]	0.757	0.54 [0.27–1.33]	

HDL: high density lipoprotein; LDL: low density lipoprotein; HOMA-IR: homeostatic model assessment of insulin resistance; QUICKI: quantitative insulin-sensitivity check index.

**Table 3:** Associations of genotypes with PON1 and oxidative stress markers.

Characteristics	PON1 rs662				PON1 rs854560				Overall
	QQ192	Q192R	192RR	*P* value	55MM	L55M	LL55	*P* value	
*n*	53 (14.3)	191 (51.6)	126 (34.1)		246 (66.5)	114 (30.8)	10 (2.7)		370
Men, *n* (%)	12 (22.6)	49 (25.7)	33 (26.2)	0.878	66 (26.8)	27 (23.7)	1 (10)	0.429	94
Diabetes, *n* (%)	17 (32.1)	67 (35.1)	28 (22.2)	0.049	71 (28.9)	36 (31.6)	5 (50.0)	0.338	112
Hypertension, *n* (%)	31 (58.5)	100 (52.4)	56 (44.4)	0.177	124 (50.4)	56 (49.1)	7 (70.0)	0.447	187
PON1 conc (*µ*g/mL)	104 [94–117]	101 [78–112]	94 [71–111]	0.015	98 [74–113]	98 [81–111]	105 [95–112]	0.429	98 [77–112]
PONase (U/L)	130 [93–182]	189 [146–222]	185 [144–222]	<0.0001	183 [132–222]	185 [132–220]	119 [96–160]	0.059	183 [130–220]
AREase (kU/L)	97 [72–119]	108 [85–133]	108 [84–132]	0.346	104 [82–132]	108 [83–133]	125 [120–137]	0.252	108 [83–132]
FRAP (*µ*M)	651 [526–754]	702 [541–832]	677 [559–831]	0.381	677 [543–840]	677 [547–763]	674 [567–755]	0.827	677 [545–817]
TEAC (nM)	1206 [837–1442]	1298 [946–1632]	1338 [971–1644]	0.053	1299 [934–1645]	1268 [945–1581]	1303 [1237–1330]	0.774	1289 [941–1626]
Ox-LDL (ng/mL)	3791 [2227–6155]	4695 [3361–6009]	4142 [2972–5298]	0.029	4281 [3184–5622]	4602 [3049–5842]	2682 [1463–6314]	0.501	4284 [3082–5719]
TBARS (nM)	1840 [942–3042]	2424 [1844–3148]	2223 [1598–2978]	0.006	2298 [1640–3128]	2282 [1625–3042]	1114 [950–2822]	0.221	2282 [1622–3116]

PON1: Paraoxonase 1; PONase: paraoxonase activity; AREase: arylesterase activity; FRAP: ferric reducing ability of plasma; TEAC: Trolox equivalent antioxidant capacity; Ox-LDL: oxidized LDL; TBARS: thiobarbituric acid reactive substances.

**Table 4:** Regression coefficients from multiple robust linear models for the prediction of indices of PON1 and antioxidant status by PON1 polymorphisms, accounting for the potential effect of sex, age, diabetes, and adiposity.

Gene	Model	PON1		PONase		AREase		FRAP		TEAC		Ox-LDL		TBARS	
		*β*	*P*	*β*	*P*	*β*	*P*	*β*	*P*	*β*	*P*	*β*	*P*	*β*	*P*
PON1 Q192 (rs662)	Alone	6.84	0.002	−18.33	0.002	−3.11	0.266	−15.68	0.299	−74.92	0.061	39.21	0.804	−42.38	0.621
	+ Age	6.18	0.006	−18.31	0.001	−2.92	0.312	−14.98	0.328	−68.15	0.096	34.14	0.814	−79.42	0.346
	+ sex	5.61	0.003	−17.99	0.0006	−3.44	0.166	−14.41	0.346	−66.78	0.080	41.48	0.777	−83.18	0.393
	+ BMI	5.38	0.008	−15.74	0.0009	−2.42	0.339	−10.41	0.471	−64.51	0.075	−54.69	0.711	−100.98	0.211
	+ Diabetes	5.82	0.001	−15.41	0.002	−0.77	0.759	−9.54	0.485	−51.33	0.121	−142.46	0.348	−109.75	0.152
	+ Diabetes^*^Q192R	3.79	0.08	−14.99	0.007	−2.94	0.291	−14.50	0.359	−40.30	0.340	−155.24	0.354	−87.90	0.321
Diabetes^*^Q192		11.32	0.007	2.09	0.848	4.85	0.366	22.86	0.472	−49.17	0.553	122.96	0.709	−138.59	0.438
PON1 L55 (rs854560)	Alone	1.76	0.518	−5.37	0.455	3.17	0.361	−21.31	0.274	−31.05	0.534	−184.80	0.314	−64.68	0.529
	+ Age	0.82	0.758	−5.39	0.461	3.22	0.376	−21.36	0.277	−30.42	0.541	−183.27	0.322	−91.05	0.355
	+ sex	1.05	0.508	−6.62	0.338	2.49	0.474	−19.21	0.354	−29.75	0.528	−156.72	0.380	−93.82	0.327
	+ BMI	2.65	0.235	−5.86	0.340	5.10	0.155	−11.71	0.523	−18.32	0.687	−220.02	0.222	−104.31	0.303
	+ Diabetes	2.77	0.217	−6.36	0.277	5.00	0.104	−12.95	0.492	−9.50	0.825	−256.49	0.154	−106.93	0.261
	+ Diabetes^*^L55M	3.03	0.278	−5.88	0.467	5.99	0.109	−24.85	0.240	−17.61	0.745	−395.49	0.075	−222.50	0.062
Diabetes^*^L55		−0.69	0.883	−5.58	0.682	−10.34	0.119	41.03	0.266	21.27	0.775	994.86	0.013	391.84	0.058
PON1 Q192 (rs662)	PON1 L55	7.13	0.002	−18.34	0.002	−4.01	0.158	−13.00	0.418	−73.65	0.077	77.21	0.635	−32.88	0.716
	+ Q192^*^L55	7.83	0.005	−15.89	0.018	−2.77	0.403	−19.55	0.297	−81.54	0.092	169.29	0.350	−44.91	0.683
PON1 L55 (rs854560)	PON1 Q192	−1.02	0.705	−0.05	0.994	4.42	0.215	−17.81	0.379	−6.39	0.902	−212.36	0.304	−57.23	0.615
	+ Q192^*^L55	2.88	0.544	11.71	0.377	8.47	0.183	−37.88	0.287	−31.84	0.728	65.66	0.849	−98.36	0.638
Q192^*^L55		−3.30	0.435	−10.16	0.339	−3.93	0.449	22.32	0.449	25.11	0.737	−282.13	0.326	40.08	0.818

BMI: body mass index; PON1: Paraoxonase 1; PONase: paraoxonase activity; AREase: arylesterase activity; FRAP: ferric reducing ability of plasma; TEAC: Trolox equivalent antioxidant capacity; Ox-LDL: oxidized LDL; TBARS: thiobarbituric acid reactive substances.
